# Arrhythmic risk biomarkers for the assessment of drug cardiotoxicity: from experiments to computer simulations

**DOI:** 10.1098/rsta.2010.0083

**Published:** 2010-06-28

**Authors:** A. Corrias, X. Jie, L. Romero, M. J. Bishop, M. Bernabeu, E. Pueyo, B. Rodriguez

**Affiliations:** 1 Oxford University Computing Laboratory, Wolfson Building, Parks Road, Oxford OX1 3QD, UK; 2 Instituto de Investigación Interuniversitario en Bioingeniería y Tecnología Orientada al Ser Humano, 6 Universidad Politécnica de Valencia (I3BH ), Valencia, Spain; 3 Instituto de Investigación en Ingeniería de Aragón (I3A), Universidad de Zaragoza, Saragossa, Spain

**Keywords:** cardiac electrophysiology, biomarkers, modelling, electrocardiogram

## Abstract

In this paper, we illustrate how advanced computational modelling and simulation can be used to investigate drug-induced effects on cardiac electrophysiology and on specific biomarkers of pro-arrhythmic risk. To do so, we first perform a thorough literature review of proposed arrhythmic risk biomarkers from the ionic to the electrocardiogram levels. The review highlights the variety of proposed biomarkers, the complexity of the mechanisms of drug-induced pro-arrhythmia and the existence of significant animal species differences in drug-induced effects on cardiac electrophysiology. Predicting drug-induced pro-arrhythmic risk solely using experiments is challenging both preclinically and clinically, as attested by the rise in the cost of releasing new compounds to the market. Computational modelling and simulation has significantly contributed to the understanding of cardiac electrophysiology and arrhythmias over the last 40 years. In the second part of this paper, we illustrate how state-of-the-art open source computational modelling and simulation tools can be used to simulate multi-scale effects of drug-induced ion channel block in ventricular electrophysiology at the cellular, tissue and whole ventricular levels for different animal species. We believe that the use of computational modelling and simulation in combination with experimental techniques could be a powerful tool for the assessment of drug safety pharmacology.

## 1. Introduction

Anti-arrhythmic drugs (classes I, III and IV) are routinely used to treat heart rhythm disorders by directly interfering with cardiac ion channel activity. However, as stated in a review of class III anti-arrhythmic drugs by MacNeil (1997), ‘proarrhythmia is a concern for all patients taking anti-arrhythmic drugs’. For example, class III anti-arrhythmic drugs prevent arrhythmias by prolonging action potential duration (APD). However, excessive action potential (AP) prolongation caused by a high dosage of class III anti-arrhythmic drugs is suspected to be the cause of a variety of forms of triggered activity at the cellular level—including early after depolarizations (EAD)—that may degenerate into life-threatening forms of arrhythmia. Moreover, pro-arrhythmia is not only an issue related to anti-arrhythmic drugs, but non-cardiac drugs can also unintentionally interfere with cardiac electrophysiology and pose risks of arrhythmogenicity. Thus, cardiac toxicity is a major concern for the pharmaceutical industry, regulatory agencies and society and represents a huge socio-economic impact. Improvements in the assessment of safety pharmacology are therefore urgent to aid in identifying cardiotoxic compounds as early as possible in the drug development process.

Regulatory agencies point at the prolongation of the interval between the Q wave and the T wave (QT interval) as the main clinically proven electrocardiogram (ECG) biomarker for drug safety. Preclinically, AP prolongation and human ether-a-go-go (hERG) block would also lead to the abandonment of the compound from further development. It is, however, well recognized that a thorough QT/QT_c_ (where QT_c_ is the QT interval corrected for the heart rate) study alone is inadequate for assessment of drug-induced cardiac toxicity owing to the poor correlation between QT prolongation and occurrence of torsade de pointes (TdP; Shah & Hondeghem 2005). In addition, it has also been extensively documented that AP prolongation or hERG block are not necessarily related to increased arrhythmic risk. Thus, a large body of research has been directed at identifying new biomarkers of drug cardiotoxicity. For instance, the TRIaD concept (i.e. triangulation of AP, reverse use dependence of the drug, beat-to-beat instability and spatial dispersion of repolarization) suggests that QT prolongation in the presence of TRIaD preferentially leads to TdP, while QT prolongation without TRIaD may be anti-arrhythmic. Thus, new ECG biomarkers obtained by extracting TRIaD from ECG, either alone or combined with the QT/QT_c_ interval, may provide a better drug safety assessment than the QT/QT_c_ interval alone. Among steady-state morphological and dynamic repolarization parameters, the most probable ECG equivalents of TRIaD have been suggested to be T-wave changes (T), QT/RR slope (R), QT variability (I) and T-peak to T-end (T_p_T_e_) interval (D; Hondeghem 2006; Antzelevitch *et al.* 2007).

Identification of new and efficient biomarkers of drug cardiotoxicity requires a deep understanding of the mechanisms of drug-induced cardiac arrhythmias. These mechanisms are often multi-scale, spanning from multiple drug-induced alterations in ion channels to whole organ properties such as propagation dynamics, and their investigation using solely experimental techniques offers important limitations. Computational modelling and simulation have been extensively used in the field of cardiac electrophysiology, and they represent promising tools for the improvement of the safety pharmacology assessment process and the identification of new biomarkers of drug cardiotoxicity. The goal of the present paper is twofold. Firstly, a literature review is performed on biomarkers for the evaluation of drug-induced arrhythmic risk from the ionic to the ECG levels. Then we illustrate the use of state-of-the-art computational modelling and simulation techniques for the simulation of drug-induced effects on cardiac electrophysiology and on specific biomarkers proposed in the literature.

## 2. Biomarkers of drug-induced arrhythmic risk

A large body of research has provided insight into the impact that alterations in specific ion channel properties has at the cellular, tissue and ECG levels. These investigations have resulted in the identification of a number of biomarkers, which could be key to the diagnosis of pathological pro-arrhythmic states. Pro-arrhythmic mechanisms can be related to alterations in ion channel properties caused by drugs, mutations and diseases, usually involving cardiac sodium, potassium and/or calcium channels. In the following sections, we review the main biomarkers identified for each of the ionic currents, providing examples of the impact of specific anti-arrhythmic or pro-arrhythmic drugs on those biomarkers.

### (a) Sodium channels

*SCN5A*-encoded Na^+^ channels have been known to be expressed in cardiac myocytes for more than three decades (Kohlhardt *et al.* 1972). Two distinct components of the Na^+^ current have been identified in cardiac myocytes: a transient component (*I*_Na(T)_) and a persistent component (*I*_Na(P)_, also termed *I*_Na(late)_). Whether these two currents are produced by the same channel or by different isoforms remains an open question (see Saint (2008) for a review and further references supporting each of the two theories). From a biophysical point of view, *I*_Na(T)_ is activated following membrane depolarization, inactivates quickly and is responsible for the upstroke phase of the cardiac AP as well as for the entrainment system between neighbouring cells that guarantees a proper conduction of the electrical stimulus. *I*_Na(P)_ differs from *I*_Na(T)_ primarily owing to different inactivation properties, almost absent for *I*_Na(P)_, but also for the slightly different steady-state activation kinetics (20 mV more negative for *I*_Na(P)_). The two currents also differ in tetrodotoxin sensitivity (Saint *et al.* 1992).

Class I anti-arrhythmic drugs are known to alter Na^+^ channel properties, resulting in depressed maximal rate of rise of cardiac APs, slowing of conduction velocity and alterations in refractoriness. Class I drugs have been traditionally subdivided into three categories—Ia, Ib and Ic—according to their kinetics of action (intermediate, fast and slow, respectively; Trevor & Katzung 2003) and also their different effects on the effective refractory period (ERP): class Ib drugs markedly depressed ERP (and shortened APD), whereas Ic drugs had minor effects on ERP and the Ia subgroup moderately prolonged ERP (and APD; Campbell 1983).

Quinidine, flecainide and lidocaine are examples of class Ia, Ib and Ic drugs, respectively, that have shown pro-arrhythmic potential as described below.

Quinidine is a class Ia drug, used to treat atrial and ventricular fibrillation. It has been shown to block both components of the sodium current (binding to its open state) and to cause a prominent reduction in upstroke velocity (Salata & Wasserstrom 1988). However, it also blocks the calcium current and a variety of potassium conductances. Its class III effects on *I*_K*r*_ have been suggested as the reason for its pro-arrhythmic potential (Yang & Roden 1996). Quinidine was found to increase ERP in a rate-independent manner in humans (Rosenheck *et al.* 1990). Interestingly, quinidine was found to be pro-arrhythmic only at low concentrations and safe at higher concentrations. Wu *et al.* (2008) proposed that an explanation can be found in the concomitant block of both *I*_K*r*_ and *I*_Na(P)_ by quinidine at higher concentrations (the IC_50_ was found to be 4.5 and 12 μM for *I*_K*r*_ and *I*_Na(P)_ blocks, respectively).

**Figure 1.**
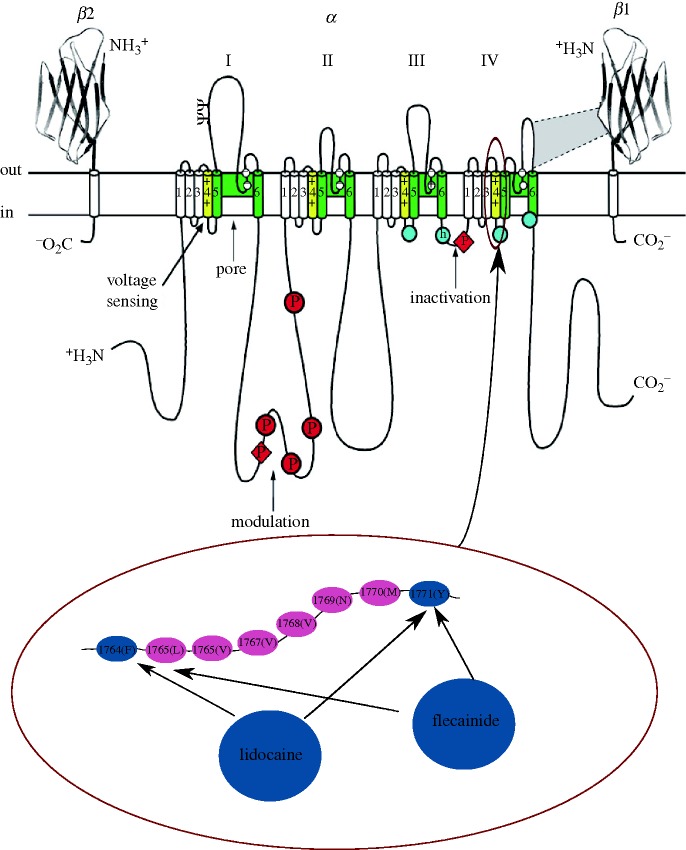
Schematic of a Na^+^ channel and drug-binding sites. The four domains of the pore-forming *α* subunit are shown together with two *β* sheets (*β*1 and *β*2). Each domain is composed of six segments (S1–S6). The pore-lining segments are shown in green (S5–S6), while the voltage-sensitive (S4) segments are shown in yellow. A part of the amino acid sequence in the S4 segments of the fourth domain is shown enlarged in the lower panel. The particular amino acids in positions 1764 and 1771 are shown in blue as they are believed to be involved in the binding of anti-arrhythmic drugs such as lidocaine and flecainide. Adapted from fig. 2 of Schauer & Catterall (2006).

Lidocaine (a local anaesthetic belonging to class Ib anti-arrhythmic drugs) was first described as a cardiac *I*_Na_ blocker with possible anti-arrhythmic effects by Bean *et al.* (1983). Lidocaine displayed affinity to the inactivated state of the channel (Liu *et al.* 2003) and appears to alter the movement of the S4 segment in the IV domain of the ion channel (Sheets & Hanck 2003) where residues at positions 1764(F) and 1771(Y) have been found to be implicated in the binding (Ragsdale *et al.* 1996; figure 1). The effect on the macroscopic current is a lower peak in the gating charge/voltage (QV) relationship. Boltzmann fits also showed a lower half-activation value and a bigger slope factor in the presence of lidocaine compared with control (Hanck *et al.* 2000). In healthy canine hearts, lidocaine slowed conduction velocity in a rate-dependent manner (no effect at 1000 ms pacing cycle length, 13–17% decrease at 200 ms pacing cycle length (Anderson *et al.* 1990)). In a recent review, Singh & Patrick (2007) classified lidocaine as having no effect (or minor shortening) on ERP, confirming early findings by Olsson *et al.* (1975) in which a correlation between ERP changes and administration of lidocaine could not be found. In ΔKPQ mutant Na^+^ channels (increased persistent *I*_Na(late)_ compared with wild type) expressed in HEK cells, lidocaine blocked the late Na^+^ current (*I*_Na(late)_) more than the peak (*I*_Na(T)_) (An *et al.* 1996).

Flecainide is an example of a class Ic drug that binds to the Na^+^ channel in its open state. Although it shares the same molecular receptor as lidocaine, it appears to reach it via an intracellular pathway rather than extracellularly (Liu *et al.* 2003). However, Liu *et al.* (2002) have shown that channel opening is necessary but not sufficient for stable drug binding and indicated the inactivation that follows channel opening as a critical process. At the tissue level, flecainide has been shown to slow conduction velocity in a heterogeneous fashion (35% decrease in the right ventricle and 29% decrease in the left ventricle; Veeraraghavan & Poelzing 2008) but has also been shown to reduce QT prolongation in some forms of long QT syndrome.

At the ECG level, it is well reported that administration of drugs that block *I*_Na(T)_ can induce (Brugada-type) ST-segment elevation (Junttila *et al.* 2008). In addition, macroscopic T-wave alternans (TWA), i.e. beat-to-beat variations in T-wave amplitude (Tamp), morphology or polarity, have reportedly been closely related to a high inducibility of ventricular arrhythmia after administration of pilsicainide in patients with Brugada syndrome (Morita *et al.* 2003; Tada *et al.* 2008). ST elevation of no less than 0.2 mV and late potentials have also been shown to be much more significant indices for risk stratification than QT prolongation in patients with a Brugada-type ECG (Ajiro *et al.* 2005; Ikeda *et al.* 2005).

Transmural dispersion of repolarization has been found to increase significantly after administration of veratridine, which leads to type 3 of long QT (LQT) syndrome (LQTS) owing to augmented *I*_Na(late)_ (Milberg *et al.* 2005). Being considered as electrocardiographic counterparts of dispersion of repolarization (Benatar *et al.* 2002; Antzelevitch *et al.* 2007), T_p_T_e_ and the ratio between T_p_T_e_ and Q-onset to T-peak interval (T_p_T_e_/QT_p_) may be useful biomarkers for monitoring cardiac safety of drugs that can increase late sodium current. It has been shown in patients with acquired (pharmaceutical-induced) LQTS that T_p_T_e_/QT_p_ was a better predictor of TdP than QT_c_ and its value of 0.28 in the V5 lead was the cut-off point for occurrence of TdP (Yamaguchi *et al.* 2003).

### (b) Potassium channels

The delayed rectifier K^+^ channels are responsible for the repolarization phase of the cardiac AP. The *KCNH2*-encoded hERG protein constitutes the pore-forming subunit of the rapid component of the delayed rectifier K^+^ channels (*I*_K*r*_) expressed in ventricular myocytes (Sanguinetti *et al.* 1995). Regulatory subunits (MiRP peptides encoded by the *KCN1* gene) are believed to co-assemble with hERG to constitute the complete *I*_K*r*_ ion channel (see Tamargo *et al.* (2004) for chromosomal locations and a more in-depth review of the K^+^ channels, genotype). During a cardiac AP, *I*_K*r*_ is activated during the plateau phase (Zhou *et al.* 1998) and is responsible for the repolarization of the transmembrane potential.

In addition, the slow component of the delayed rectifier current (*I*_K*s*_) is expressed in cardiac ventricular myocytes and contributes to the AP repolarization phase. The genotype of the pore-forming subunit has been elusive for many years and is now believed to be constituted by the assembly of KCNQ1 (also termed KvLQT1) and KCNE (also termed minK or *I*_*s*K_) gene products (Sesti & Goldstein 1998; Suessbrich & Busch 1999). In healthy tissues, full block of *I*_K*s*_ failed to have a significant impact on the APD in ventricular myocytes of rabbits (only 4% mean prolongation in the presence of *I*_K*s*_ blocker chromanol 293B (Lengyel *et al.* 2001)), dogs (a frequency-independent 7% increase with the same blocker (Varro *et al.* 2000)) and human myocytes (APD changes less than 12 ms (Jost *et al.* 2005)). However, chromanol 293B had a significant impact on pharmacologically lengthened APD in canine myocytes, suggesting a primary role of *I*_K*s*_ in pathological conditions (Varro *et al.* 2000). Similarly, in human myocytes, blockade of *I*_K*s*_ by HMR-1556 caused significant APD prolongation when repolarization reserve was reduced (by *I*_K*r*_ block) and sympathetic activation was present (Jost *et al.* 2005). In an experimental model of rabbit with AV block-induced long QT interval and TdP, *I*_K*s*_ (among other currents) was found to be reduced by 50 per cent (Tsuji *et al.* 2002). Cheng & Kodama (2004) also suggest that *I*_K*s*_ (and *I*_K*r*_) is likely to contribute to arrhythmogenesis in diseased hearts via the spatially heterogeneous prolongation of APD.

*I*_K*r*_ is considered to be the most widely targeted K^+^ channel linked to potential arrhythmogenicity. The evaluation of the effects of drugs on hERG-encoded ion channels is considered of primary importance in preclinical tests. In fact, a relationship between blockage of *I*_K*r*_ at therapeutic concentrations and risk of onset of TdP through delayed repolarization is now generally accepted in drug evaluation (Gintant *et al.* 2006). For example, cisapride, a gastrointestinal prokinetic agent (Carlsson *et al.* 1997), and terfenadine, a non-sedating antihistamine (Roy *et al.* 1996), were both withdrawn from the market between 1997 and 2000 owing to their hERG block effects.

The biophysical interactions between class III drug compounds and the ion channel protein have been the subjects of extensive investigation. It has been suggested that multiple aromatic rings on the inner surface of the pore, a rather unique characteristic of hERG-encoded channels, are responsible for the high affinity of drugs with similar aromatic rings in their structure (Vandenberg *et al.* 2001). Methanesulfonanilides (e.g. E-4031, dofetilide, MK-499) have been shown to bind to the open state of the channel (IC_50_∼130 nM) without altering any of its kinetic properties (Spector *et al.* 1996). However, Herzberg *et al.* (1998) were able to confer E-4031 sensitivity to the E-4031-insensitive M-eag channels by transplanting the hERG inactivation domain into it, suggesting a fundamental role of channel inactivation to the stability of drug binding. Similarly, Numaguchi *et al.* (2000) found that the affinity of dofetilide for hERG was nearly eliminated in a non-inactivating hERG double mutant (G628C–S631C) compared with wild-type.

The rate dependency of drug binding to hERG channels has been suggested as one of the factors that lead some class III anti-arrhythmic drugs to be ineffective or even become pro-arrhythmic. Delayed repolarization is thought to be beneficial during ventricular tachycardia because of the subsequent increase in the refractory period, which also prevents the formation of potentially fatal re-entrant circuits (re-entrant arrhythmia). However, many *I*_K*r*_ blockers, which prolong APD, are known to have a negative correlation between their potency and heart frequency, i.e. they do not work as well at higher frequencies (which is exactly when they are needed) and are more potent at lower frequencies, exposing the heart to extremely prolonged APD and risk of TdP after episodes of bradycardia (Hondeghem & Snyders 1990; Varro *et al.* 2004; Bnsz *et al.* 2009).

For example, the development of d-sotalol, a *I*_K*r*_ blocker, was abandoned because of poor clinical results and increased mortality compared with placebo in patients with a defective left myocardial function (ejection volume less than 40% and/or history of myocardial infarction (Waldo *et al.* 1996)). Similarly, cases of TdP have been reported during administration of dofetilide (Moller 1996). Interestingly, the ability of dofetilide to increase ERP was found to be decreased at higher heart rates and increased at low heart rates (Bauer *et al.* 1999), which could be related to drug-induced increased pro-arrhythmic risk as described above.

Nevertheless, block of hERG-encoded *I*_K*r*_ does not necessarily imply delayed repolarization and potential arrhythmogenesis. Verapamil, for example, is a vasodilatory and anti-arrhythmic drug that has been reported to block *I*_K*r*_ ion channels at therapeutic concentrations (Duan *et al.* 2007). The lack of an AP prolongation effect is thought to be due to the concomitant blockage of L-type Ca^2+^ channels by verapamil at similar concentrations and a subsequently restored balance between hyperpolarizing and depolarizing currents during the AP plateau phase (Gintant *et al.* 2006).

The involvement of abnormal *I*_K*s*_ behaviour in arrhythmogenicity was suggested by Wang *et al.* (1996) and Chen *et al.* (2003). They identified several types of genetic mutations (mostly missense mutations) in the *KvLQT1* gene in families with congenital LQTS (type 1) and increased susceptibility to cardiac tachyarrhythmias and sudden death. *I*_K*s*_ has been recently evaluated as a potential target for anti-arrhythmic drugs after unsatisfactory results of clinical trials with *I*_K*r*_ blocking agents such as d-sotalol (Gerlach 2003). Three main compounds have shown the ability of selectively blocking *I*_K*s*_: the above-mentioned chromanol 293B, benzodiazepines and benzamides (Gerlach 2003). Although KCQN1 appears to be the main molecular target, it has been shown that KCNE allosterically facilitates drug binding resulting in a 6–100-fold increase in affinity (Busch *et al.* 1997; Tamargo *et al.* 2004). Benzodiazepine L-7 blocks KCNQ1 channels by binding to the S6 protein domain; normalized *I*–*V* curves and activation kinetics were not affected by the presence of L-7, suggesting that the block is voltage independent (Seebohm *et al.* 2003). The beneficial effects of chromanol were evaluated in the intact canine heart and it was found that blockage of *I*_K*s*_ produced a spatially uniform increase in ERPs that, unlike blockage of *I*_K*r*_ by dofetilide, became more pronounced at higher heart rates (Bauer *et al.* 1999). This is thought to preserve the heart from re-entrant arrhythmias (Wellens *et al.* 1984). Similarly, benzodiazepines (L-768 673 compound) were able to reduce ventricular fibrillation and incidence of arrhythmias in a canine experimental model of recently infarcted heart (Lynch *et al.* 1999).

Administration of drugs that block *I*_K*r*_ and/or *I*_K*s*_ can change steady-state behaviour of repolarization reflected in T-wave morphology, time interval and T-wave vector loop morphology. In terms of T-wave morphology, increased JT area (total area of the T-wave) has been reported (Thomsen *et al.* 2006*a*,*b*), representing increased interventricular dispersion of repolarization (van Opstal *et al.* 2002). T-wave area-based parameters have been shown to be indices as effective as QT interval for identification of sotalol-induced repolarization changes (Couderc *et al.* 2003). Moreover, such morphological measurements take into account not only the morphology of repolarization but also the entire process of repolarization. They have been shown to be more stable and thus more reliable than manual QT measurement (Couderc *et al.* 2003). Decreased Tamp, increased U-wave amplitude, increased ratio of U-wave to T-wave amplitude, and increased incidence of T-wave notching have also been observed following the use of *I*_K*r*_/*I*_K*s*_ blockers (Houltz *et al.* 1999; Gbadebo *et al.* 2002; Thomsen *et al.* 2006*a*,*b*). Recent studies conducted by researchers in Denmark have demonstrated that an overall morphology score, which evaluates the asymmetry, notch and flatness of the T-wave, could discriminate patients with *LQT2* (hERG) mutations from normal controls (Andersen *et al.* 2007). The score has also been shown to be a more sensitive measure of repolarization changes induced by an *I*_K*r*_-inhibiting compound (Lu 35-138) than the QT/QT_c_ interval (Graff*et al.* 2008). Changes in steady-state repolarization are also reflected in changes in time intervals, such as increased T_p_T_e_ and T_p_T_e_/QT_p_ (Benatar *et al.* 2002; Liu *et al.* 2006; Thomsen *et al.* 2006*a*,*b*; Gallacher *et al.* 2007), which represents increased transmural dispersion of repolarization (Benatar *et al*. 2002; Antzelevitch *et al.* 2007) as mentioned previously. T_p_T_e_/QT_p_ has been suggested to be a better biomarker than QT and T_p_T_e_ in assessment of pro-arrhythmic effects of *I*_K*r*_-blockers (Liu *et al.* 2006), while T_p_T_e_ has been shown to be a better indicator of spontaneous TdP induced by *I*_K*s*_-blockers than QT (So *et al.* 2008). In terms of T-loop morphology, *I*_K*r*_-blocker-induced changes in vectorcardiograms derived from conventional 12-lead ECG have been investigated, including increased early and late repolarization duration measured from the T loop (Couderc *et al.* 2006, 2008). Such morphology parameters have been shown to be better in detecting the existence of an *I*_K*r*_-blocker than QT_c_ interval (Couderc *et al.* 2008).

*I*_K*r*_/*I*_K*s*_ blockers can also lead to changes in the dynamic behaviour of repolarization, such as decreased Tamp/RR ratio, decreased QT/TQ ratio (Fossa *et al.* 2007) and increased slope of the QT/RR relationship (Lande *et al.* 1998; Couderc *et al.* 2003; Smetana *et al.* 2004). Variation of QT also increases, which indicates increased temporal dispersion of repolarization, and can be assessed by the QT variability index (evaluated over several beats (Berger *et al.* 1997)) or by beat-to-beat QT variability (evaluated from Poincare plots (van der Linde *et al.* 2005)). The latter has been shown to be superior to QT prolongation for predicting occurrence of TdP induced by *I*_K*r*_-blocking drugs (Thomsen *et al.* 2004, 2006*a*,*b*).

Microvolt TWA has been observed after administration of pentamidine (Kroll & Gettes 2002), which can prolong the QT interval by reducing hERG expression (Cordes *et al.* 2005), and thus proposed to be potentially useful for identification of patients who exhibit higher risk for lethal arrhythmias. Spectral analytical method is the most common method for TWA detection from stationary ECG recordings (Bloomfield *et al.* 2002). Non-spectral technique, such as the modified moving average beat analysis, has been used for Holter recordings (Verrier *et al.* 2005).

### (c) Calcium channels

L-type Ca^2+^ channels are commonly expressed in mammalian cells of excitable and non-excitable tissues by the *CACNA1C* gene. They have traditionally been classified by their sensitivity to dihydropyridine-based compounds (e.g. nifedipine) and constitute one of the most important Ca^2+^ entry pathways into the cell. The ubiquitous role of Ca^2+^ in cellular pathophysiology underlies the implication of L-type Ca^2+^ channels in a variety of diseases of diverse nature. In the heart, L-type Ca^2+^ channel abnormalities have been linked to ventricular arrhythmias, impaired excitation–contraction coupling leading to heart failure, as well as atrial fibrillation (see Bodi *et al.* 2005).

Three main classes of drugs are known to interfere with L-type Ca^2+^ channel activity: phenylalkylamines (e.g. verapamil), benzothiazepines (e.g. diltiazem) and dihydropyridines (e.g. nifedipine and Bay K 8644 (Hockerman *et al.* 1997)). Compounds belonging to all three classes directly interact with the IIIS6 and IVS6 transmembrane segments of the *α*_1_ subunit of the channel in a voltage and state-dependent fashion that was found to be consistent with the modulated receptor model initially proposed for local anaesthetics with Na^+^ channels (McDonald *et al.* 1984). While phenylalkylamines and benzothiazepines are for the most part channel blockers, different compounds belonging to the dihydropyridines class may be either agonists or antagonists. A link between L-type Ca^2+^ channels agonist dihydropyridines and cardiac arrhythmias was first proposed by January & Riddle (1989). L-type Ca^2+^ channel agonist Bay K 8644 was shown to induce EADs from an average take-off potential of −34 *mV*. In the presence of Bay K 8644, L-type Ca^2+^ current was augmented and the peak of the *I*–*V* curve was shifted to more negative potentials indicating interference with the kinetic properties of the ion channel. Furthermore, it was pointed out that time-dependent properties such as recovery from inactivation could be of primary importance for the onset of EADs. These findings were further substantiated by a later modelling study by Zeng & Rudy (1995), where the effects of a β-adrenergic agent (isoproterenol) on L-type Ca^2+^ channels were simulated and incorporated into a whole-cell Luo–Rudy model. In particular, owing to experimental results of patch clamp experiments on isolated canine myocytes by Priori & Corr (1990), the authors modified the normal equation of the Hodgkin and Huxley formulation of *I*_CaL_ by increasing the inactivation time constant (13%) and the maximal conductance (fivefold increase). Results of whole cell simulations under such conditions showed the ability of β-adrenergic agents to initiate an EAD, confirming the potential implication of L-type Ca^2+^ channels in the arrhythmogenic process. Similar conclusions regarding the involvement of *I*_CaL_ were reached by Viswanathan & Rudy (1999) in a simulation study of pause-induced EADs.

The arrhythmogenic effect of stimulating L-type Ca^2+^ channels was also found to be potentiated by acetylcholine in guinea pig ventricular myocytes (Song *et al.* 1998). The implication of *I*_CaL_ in the arrhythmogenic process was further studied in a rabbit experimental model with chronic atrioventricular block (AVB; Tsuji *et al.* 2002). In such animals, the incidence of acquired QT prolongation and TdP was significantly higher than in similar experiments with dogs. The electrophysiological properties of L-type Ca^2+^ channels were found to be altered. In particular, the steady-state activation curve of *I*_CaL_ was shifted towards the negative direction whereas the inactivation kinetics were unaltered. These results are, again, in contrast with previous experiments on dogs that seemed to imply an involvement of the inactivation kinetics of *I*_CaL_ in arrhythmogenesis.

Another link between L-type Ca^2+^ channels and cardiac arrhythmias was established by the study of patients affected by Timothy syndrome (Splawski *et al.* 2004). It was found that the origin of this multi-system disorder was a de novo missense mutation affecting the *Cav1.2* gene (one of the aliases commonly used for the *CACNA1C* gene). Patients affected by Timothy syndrome displayed a variety of symptoms affecting different tissues and organs (autism and finger syndactyly, among others). In the heart, prolonged QT interval was found in 100 per cent of the patients and ventricular tachycardia in 71 per cent. From an electrophysiological point of view, it was shown that the mutation underlying Timothy syndrome caused a loss of voltage-dependent inactivation, resulting in a gain of function of the L-type Ca^2+^ channel. Voltage clamp data were incorporated into the Luo–Rudy model and a prolonged APD was predicted.

Regarding the effect of calcium antagonists on the ECG, verapamil and diltiazem have been widely reported to prolong the PQ interval (atrioventricular conduction time) while producing no significant changes in QT/QT_c_ (Heng *et al.* 1975; Della Paschoa *et al.* 1995; Busse *et al.* 2006). QT variability, however, has been shown to increase significantly following administration of diltiazem (Yamabe *et al.* 2007).

## 3. Computational assessment of the impact of drug-induced alterations on cardiac electrophysiology: from ion channels to ECG

As illustrated in the previous section, drug-induced alterations in ionic current properties result in complex changes in cardiac electrophysiological activity, which often involve multi-scale mechanisms from the ionic to the whole organ level and exhibit important animal species differences. Over the last five decades, computational cardiac electrophysiology has developed into a mature discipline, and state-of-the-art computational models are routinely used to investigate heart rhythm mechanisms. The following sections provide a description of advanced computational tools and models developed within the Computational Biology Group at the University of Oxford, which are freely available to the scientific community. We present three simulation studies that illustrate how these tools can be used for the simulation of drug-induced effects on cardiac electrophysiology using cellular, tissue and whole ventricular models for different animal species including (and not limited to) human, rabbit and guinea pig.

### (a) Computational tools and models for heart rhythm research

Since 1960, when Denis Noble published the first cell model of the cardiac AP (Noble 1960), a large number of mathematical models of the cellular AP have been developed for different cell types (i.e. sino-atrial, Purkinje, atrial, ventricular and fibroblast) and animal species (including human, dog, rabbit, guinea pig and rat). The complexity of these models varies, but the most complex ones can include 60–80 ordinary differential equations and hundreds of parameters to describe the ionic processes underlying cardiac cellular electrophysiological activity. Most of the AP models are now available in the CellML repository (http://www.cellml.org/). The CellML (Lloyd *et al.* 2004) code for the cellular AP models can be used to conduct simulations with freely available software such as COR (http://cor.physiol.ox.ac.uk/) or can be converted from CellML code to a variety of programming languages (such as Matlab or C++) with software such as PyCml (https://chaste.comlab.ox.ac.uk/cellml/).

Simulation of cardiac electrophysiology activity using tissue or whole organ models is a computationally expensive task that requires the use of sophisticated numerical and computational techniques. The Chaste simulator is, to date, the only open source software package which can be used for the simulation of cardiac electrophysiological activity from the ionic to the ECG level. Chaste has been developed at the University of Oxford with inputs from industrial partners such as Fujitsu Laboratories Europe and the code is available from http://www.comlab.ox.ac.uk/Chaste. As described in Pitt-Francis *et al.* (2008), Chaste was developed with four main specific requirements: (i) to use state-of-the-art software engineering methods, (ii) to achieve maximum efficiency on high performance computing (HPC) platforms by using state-of-the-art numerical and computational techniques, (iii) to be freely available (including source code) to the scientific community, and (iv) to be generic enough, and not constrained to a particular application. Chaste can be used for the simulation of cardiac electrophysiological activity from the ionic to the ECG level, using any AP model available in the CellML repository and any tissue or whole organ geometry.

In the following sections we describe the use of cellular, tissue and whole ventricular models for the simulation of the effect of alterations on cellular, tissue and ECG biomarkers of arrhythmic risk. In §3*b*, we describe a recent study in which one of the most detailed human ventricular AP models was used to investigate the impact of variability in ionic current properties on cellular biomarkers of arrhythmic risk (Romero *et al.* 2009). In §3*c*,*d*, tissue and whole ventricular models are used to illustrate how the impact of ion channel block on the tissue and ECG biomarkers can be simulated using the cardiac simulator Chaste.

### (b) Impact of ion channel variability on preclinical cellular biomarkers of arrhythmic risk

In a recent study, Romero *et al.* (in press) analysed the sensitivity of the main preclinical biomarkers of arrhythmic risk to changes in transmembrane ionic current conductances and the kinetics involved in AP repolarization in humans. Different stimulation protocols were applied to the human ventricular model (ten Tusscher *et al.* 2004) to study the impact of changes in transmembrane ionic current properties on cellular electrophysiological properties related to arrhythmic risk. In particular, APD, AP triangulation, diastolic and systolic calcium levels at normal (1 Hz) and slow rates (0.5 Hz), maximum slope of the standard (slope_max,S1S2_) and the dynamic (slope_max,DYN_) restitution curves, fast and slow time constants of the APD adaptation to changes in heart rhythm (*τ*_fast_ and *τ*_slow_, respectively) and intracellular calcium and intracellular sodium concentration rate dependence were investigated. A total of 10 440 simulations were run. The simulation of 3000 ms of cellular activity in an Intel Core 2 Quad CPU 2.39 GHz 1.96 GB RAM took 1.5 s.

**Figure 2.**
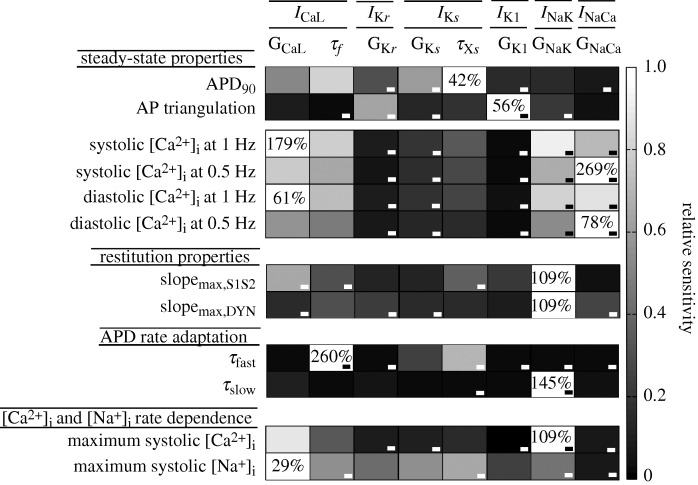
Impact of ionic current variability on cellular electrophysiological biomarkers of arrhythmic risk. Electrophysiological properties are shown in the first column and ionic current properties appear in the first row. Relative sensitivities are depicted in grey code, with white being the colour that indicates the maximum sensitivity of an electrophysiological property. *I*_CaL_, L-type calcium current; *I*_K*r*_, the rapid component of the delayed rectifier current; *I*_K*s*_, the slow component of the delayed rectifier current; *I*_*K*1_, inward rectifier potassium current; *I*_NaK_, sodium–potassium pump current; *I*_NaCa_, sodium–calcium exchanger current; G_CaL_, maximal conductance of *I*_CaL_; *τ*_*f*_, slow voltage-dependent inactivation gate time constants of *I*_CaL_; G_K*r*_, maximal conductance of *I*_K*r*_; G_K*s*_, maximal conductance of *I*_K*s*_; *τ*_*Xs*_, activation time constant of *I*_K*s*_; G_K1_, maximal conductance of *I*_K1_; G_NaK_, maximal activity of the sodium–potassium pump; G_NaCa_, maximal activity of the sodium–calcium exchanger.

The relative sensitivities of each cellular biomarker to changes in each current property found in that study are represented in figure 2 in grey scale, except for activation and fast voltage-dependent inactivation gate time constants of *I*_CaL_ and activation and inactivation gate time constants of the rapid component of the delayed rectifier current as their effects were negligible. In figure 2, the highest sensitivity of a biomarker is represented in white and its absolute value is also shown in each white box. The figure shows that changes in any repolarization current conductance and in *I*_CaL_ inactivation kinetics as well as the slow component of the delayed rectifier current (*τ*_*Xs*_) can effectively modify the APD. By contrast, AP triangulation is basically determined by inward rectifier potassium current (*I*_K1_) and *I*_K*s*_. In addition, adaptation of AP duration to rate changes, restitution properties and intracellular calcium and sodium concentrations depend on *I*_CaL_ properties and the sodium–potassium pump. As each column represents the effect of a certain ionic current modification, potential side effects of a new component could be anticipated by using this sensitivity analysis.

A similar sensitivity study was conducted using the Shannon *et al*. rabbit ventricular AP model (Romero *et al.* 2009). Our results showed that, similarly to humans, rabbit APD is moderately sensitive to changes in all repolarization currents. However, the effect of *I*_NaK_, *I*_CaL_ and *I*_K*r*_ is more relevant in rabbit myocytes. AP triangulation is strongly dependent on *I*_K1_ and *I*_K*r*_, as in humans. In addition, AP rate dependence is markedly modified by *I*_NaK_, *I*_NaCa_ and *I*_CaL_, which play a major role in this electrophysiological property in humans, but also by *I*_K*r*_ and *I*_K1_. Furthermore, intracellular Ca^2+^ and Na^+^ levels are very sensitive to *I*_NaK_ and *I*_NaCa_, which supports our results obtained in virtual human cardiomyocytes.

Therefore, this sensitivity analysis can also be used to compare the electrophysiological behaviour between different species and to anticipate discrepancies in drug effects between the experiments on different animal species (including human) and different cell types (Sanchez *et al.* 2009).

### (c) Simulation of multi-scale electrophysiological effects of ion channel block in a ventricular tissue slab

In this section, we present results of the simulation of the effect of ion channel block on cardiac electrophysiological activity in a slab of ventricular tissue obtained using the Chaste simulator. The electrical properties of the 0.45 cm edge length slab were simulated using the bidomain model. The tissue model included epicardial (0.11 cm), mid-myocardial (0.17 cm) and endocardial (0.17 cm) layers (Saucerman *et al.* 2004). In order to examine species difference in response to changes in *I*_K*r*_, simulations were conducted with membrane kinetics represented by the Mahajan–Shiferaw rabbit ventricular model (Mahajan *et al.* 2008) and the Luo–Rudy dynamic mammalian model (Faber & Rudy 2000). Transmural heterogeneities in *I*_K*s*_ and transient outward current (*I*_to_) were simulated as in previous studies (McIntosh *et al.* 2000; Gima & Rudy 2002). Homogeneous *I*_K*r*_ blockade was simulated by decreasing the maximum conductance of *I*_K*r*_ from its control value to 0 in steps of 20 per cent throughout the slab.

To ensure steady-state propagation, the slab was paced from the entire endocardial surface at a basic cycle length of 300 ms. Action potentials (APs) and pseudo-ECG during the last pacing beat were analysed. The pseudo-ECG was recorded as the extracellular unipolar potential from the centre of the outmost epicardial layer. The pseudo-ECG is obtained by assimilating the electrical activity of the heart to a single electrical dipole. This, together with the simplified geometry of a tissue slab, inevitably causes loss of information in the simulated signal when compared with an anatomically realistic solution of the forward problem of electrocardiography (Bradley *et al.* 2000). Nevertheless, certain time-dependent (e.g. QT interval) as well as morphological (e.g. ST segment elevation) features are still well represented in the pseudo-ECG signal. Interestingly, the pseudo-ECG is also used experimentally in wedge preparations (Weinberg *et al.* 2008). The computational mesh consisted of 162 000 tetrahedral elements (edge width of 0.015 cm). Simulations were run with 5 μs time steps on a four-processor computer (AMD Phenom(tm) 9600B Quad-core Processor 1.15 GHz 3.9 GB RAM). A full AP (400 ms) took 4 hours to simulate. According to the parallel speed-up reported by Pitt-Francis *et al.* (2009), the same simulation would take just under half an hour on 64 processors.

**Figure 3.**
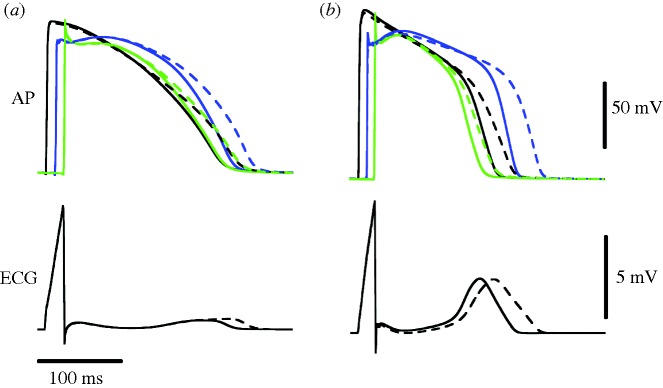
Action potentials (AP) from a representative node of each transmural layer and also the pseudo-ECG for the control (solid lines) and 100% *I*_K*r*_ blockade (dashed lines) conditions, for the rabbit (*a*) and the guinea pig (*b*) models (black line, endocardium; blue line, M cell; green line, epicardium).

**Figure 4.**
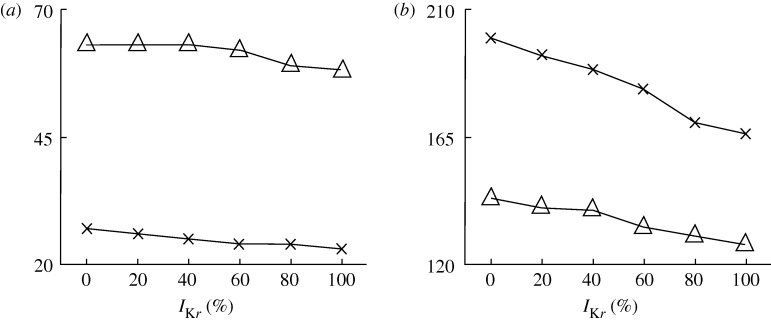
Transmural dispersion of repolarization (*a*) and QT_p_ (*b*) with varying degrees of *I*_K*r*_ blockade for the rabbit (crosses) and guinea pig (triangles) models.

Figure 3 shows the time course of the AP from a representative node of each transmural layer and also the pseudo-ECG for the control (solid lines) and 100 per cent *I*_K*r*_ blockade (dashed lines) conditions, for the rabbit (figure 3*a*) and the guinea pig (figure 3*b*) models. Clearly, in both models, *I*_r_ blockade led to prolongation of APD in all three layers and the interval between Q-wave and the peak of the T-wave (QT_p_). Figure 4 presents changes in transmural dispersion of repolarization (TDR; measured as the maximum difference in transmural APD) and QT_p_ with varying degrees of *I*_K*r*_ blockade for the rabbit (crosses) and guinea pig (triangles) models. Specifically, as the degree of *I*_K*r*_ blockade increased from 0 to 100 per cent, TDR increased gradually by 17 and 8 per cent, together with a gradual increase in QT_p_ by 20 and 13 per cent for the rabbit and guinea pig model, respectively. Here, we present simulations using two ionic specific models and we evaluate two sets of biomarkers. However, it must be noted that users could use any AP model and investigate any biomarker in addition to those described in the previous section.

### (d) Simulation of the impact of ion channel block on whole ventricular electrophysiology

**Figure 5.**
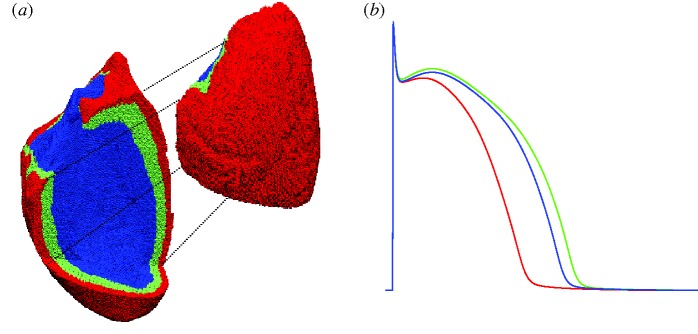
(*a*) The whole ventricular mesh. A portion of it is shifted to allow visualization of the three layers in which the wall has been subdivided: endocardial (blue), mid-myocardial (green) and epicardial (red). (*b*) Action potentials of isolated cells included in the three layers.

In this section, a rabbit ventricular model was used to simulate the impact of ion channel block on the ECG, under several conditions of tissue coupling. The propagation of the AP across the cardiac muscle was simulated by solving the monodomain equation using the Chaste simulator (Pitt-Francis *et al.* 2009). Potse *et al.* (2006) have shown that, in most cases when the extracellular potential is not of specific interest, the distribution of *V*_m_ calculated with the monodomain and bidomain equations are very similar. Having to solve one equation instead of two, the monodomain model has the advantage of reduced computational cost. Hence, although the Chaste software fully supports the solution of the bidomain equations for the whole heart, here the monodomain model was used. Assuming a constant conductivity tensor, the monodomain equation is3.1
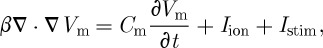

where *V*_m_ is the transmembrane potential, *C*_m_ is the membrane capacitance per unit of tissue area, *I*_ion_ is given by the equations in the Mahajan–Shiferaw model of a rabbit ventricular cell (Mahajan *et al.* 2008), *I*_stim_ is an intracellular stimulus current and *β* is a diffusion coefficient (see below). The geometry of the rabbit ventricles was reconstructed from MRI images as described in Bishop *et al.* (2010) and discretized by 3 172 910 tetrahedral elements (average distance between nodes was 250.741 μM). Transmural cellular heterogeneities were modelled by dividing the cardiac wall in epicardial, mid-myocardial and endocardial layers as shown in figure 5*a* in relative proportions of 2:3:3, respectively (Saucerman *et al.* 2004). In each of these layers, parameters for *I*_K*s*_ and *I*_to_ were scaled in order to match the experimental observations on AP duration in single-cell experiments by McIntosh *et al.* (2000) in a similar way to that proposed by Saucerman *et al.* (2004; figure 5*b*).

Endocardial activation from the Purkinje system was replicated in the whole ventricular mesh by applying an intracellular stimulus to nodes located in the apical third of the endocardial surface.

Chaste simulations were launched through a grid middleware platform (Nimrod), as described previously (Bernabeu *et al.* 2009). The monodomain equation was solved with a PDE time step of 0.01 ms and an ODE time step of 0.005 ms. Each simulation was assigned 16 processors in parallel and took about 4 h to simulate 500 ms and output results.

The unipolar pseudo-ECG (*P*) recorded at a location (*x*_0_, *y*_0_, *z*_0_) was computed as the integral of the derivative of the transmembrane potential across the heart geometry according to the equation (Baher *et al.* 2007)3.2
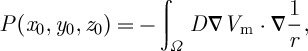

where *Ω* is the geometry under consideration (the whole heart in this case), *D* is the diffusion coefficient of the electrical medium surrounding the heart (assumed constant), *V*_m_ is the transmembrane potential and *r* is the distance between the recording electrode (*x*_0_, *y*_0_, *z*_0_) and a point (*x*, *y*, *z*) within the cardiac tissue.

**Figure 6.**
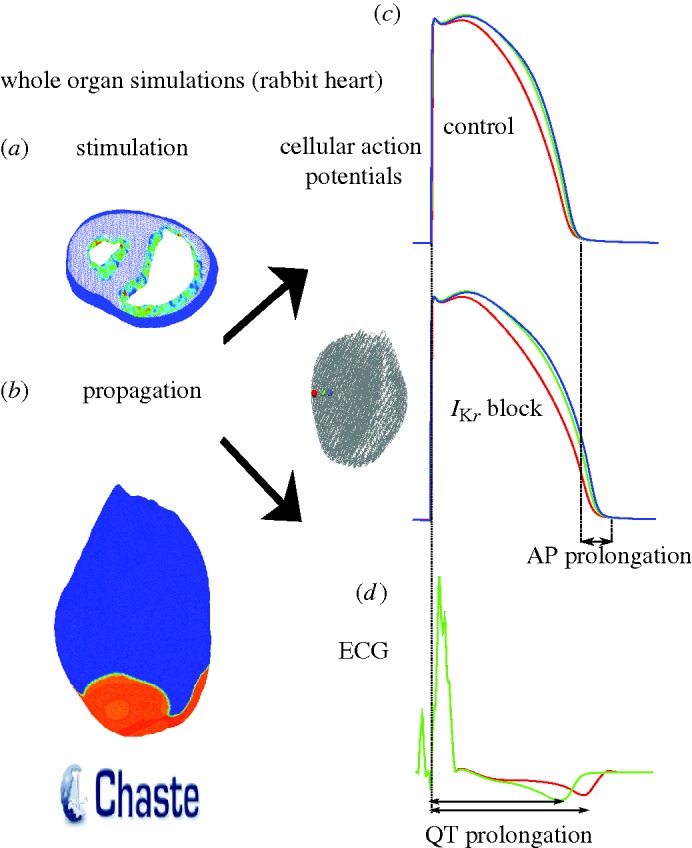
(*a*) Endocardial activation seen in a sliced whole heart. (*b*) Snapshot of AP propagation across the epicardial surface. (*c*) Representative AP traces in control and *I*_K*r*_ block conditions at nodes located in the epicardial (red line), mid-myocardial (green line) and endocardial (blue line) layers. (*d*) Pseudo-ECG computed at a distance of approximately 3 cm from the epicardium (*I*_K*r*_ block, red line; control, green line).

Figure 6*a* shows a transverse slice of the whole heart 3 ms after endocardial stimulation. The depolarizing wave reaches the epicardium and starts propagating towards the apex (figure 6*b*). Figure 6*c* shows representative APs from three nodes (location shown in the inset) under control conditions and under the effect of *I*_K*r*_ block. The differences in AP shape and duration between epicardial, mid-myocardial and endocardial layers are less marked than with a single-cell situation (figure 5*b*) owing to cell-to-cell electrotonic interactions. APD prolongation caused by *I*_K*r*_ block was 40, 39 and 40 ms in the epicardial, mid-myocardial and endocardial representative nodes, respectively. These variations in AP duration are reflected in variations in the QT interval at the ECG level (figure 6*d*) where the QT interval was prolonged by 42 ms.

**Figure 7.**
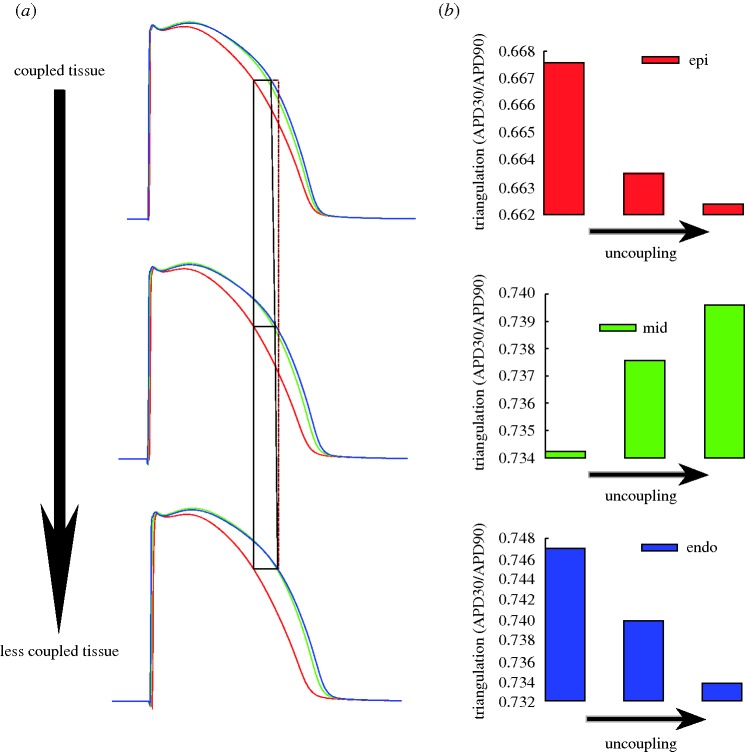
(*a*) Action potentials recorded at representative nodes in the mesh under different coupling conditions (*β*=0.428 μs in the top panel, *β*= 0.214 μs in the middle panel and *β*=0.14 μs in the bottom panel; endocardial, blue line; mid-myocardial, green line; and epicardial, red line). The differences between epicardial and endocardial APs are highlighted (red line is a straight line). (*b*) Effect of coupling on APD triangulation for epicardial, mid-myocardial and endocardial nodes.

The role of intercellular coupling in modulating transmural APD heterogeneity and QT interval was evaluated by varying the diffusion coefficient *β* in the monodomain equation. Simulations were conducted for three cases of intercellular coupling (*β*=0.428, 0.214 and 0.14 μs). The three values of intercellular coupling gave rise to propagation velocities across the tissue of 39.2, 26.7 and 19.6 cm s^−1^, respectively. Figure 7*a* shows APs recorded at representative nodes (the same as in figure 6) under different coupling conditions. As the tissue becomes less coupled the differences between epicardial, mid-myocardial and endocardial APs increase and tend to approach the isolated cell AP. The variation in AP shape due to intercellular coupling is shown in figure 7*b*, where an estimate of AP triangulation (ratio between APD_30_ and APD_90_) is shown for the three different degrees of coupling. While epicardial and endocardial cells slightly increase the value of AP triangulation, as the tissue becomes less coupled, the opposite is seen for mid-myocardial cells. Note that these trends reflect the tendency of an uncoupled tissue to behave in a similar way to isolated cells.

## 4. Conclusions

It is now well established that the development of drug-induced cardiac arrhythmia is an extremely complex and diversified pathophysiological phenomenon that involves processes at different scales, from molecular to cellular and tissue levels. Furthermore, as clinical evaluation of cardiac rhythmicity is routinely performed through the examination of ECG traces, it has become increasingly important to understand the relationships among events occurring at the nanoscale (molecular), microscale (cellular) and macroscale (ECG). The first part of this paper provides a thorough review of the biomarkers of drug-induced arrhythmic risk proposed in the literature from the ionic to the ECG level. The review presents evidence for the existence of a variety of biomarkers, the complexity of the mechanisms involved in drug-induced pro-arrhythmia and some significant animal species differences, especially in drug-induced effects on cardiac ion channels. Predicting drug-induced pro-arrhythmic risk is therefore challenging both preclinically and clinically, as attested by the rise in the cost of releasing new compounds onto the market.

Computational modelling and simulation have significantly contributed to the understanding of cardiac electrophysiology and arrhythmias over the last 40 years. The second part of this paper is aimed at demonstrating the ability of state-of-the-art computational tools to capture the multi-scale complexity of cardiac arrhythmias. Open source computational modelling software was used to simulate multi-scale effects of drug-induced ion channel block in ventricular electrophysiology at the cellular, tissue and whole ventricular levels for different animal species. Owing to its vast use in pre-clinical assessment, *I*_K*r*_ was chosen as an example for our simulations. Nevertheless, the same processes can be repeated for any molecular target and also for the evaluation of drugs acting on more than one target simultaneously. We believe that the use of computational modelling and simulation in combination with experimental techniques could be a powerful tool for the assessment of drug safety pharmacology.
